# Estimating the mutational load for cardiovascular diseases in Pakistani population

**DOI:** 10.1371/journal.pone.0192446

**Published:** 2018-02-08

**Authors:** Muhammad Shakeel, Muhammad Irfan, Ishtiaq Ahmad Khan

**Affiliations:** Jamil-ur-Rahman Center for Genome Research, Dr. Panjwani Center for Molecular Medicine and Drug Research, International Center for Chemical and Biological Sciences, University of Karachi, Karachi, Pakistan; Banaras Hindu University, INDIA

## Abstract

The deleterious genetic variants contributing to certain diseases may differ in terms of number and allele frequency from population to population depending on their evolutionary background. Here, we prioritize the deleterious variants from Pakistani population in manually curated gene list already reported to be associated with common, Mendelian, and congenital cardiovascular diseases (CVDs) using the genome/exome sequencing data of Pakistani individuals publically available in 1000 Genomes Project (PJL), and Exome Aggregation Consortium (ExAC) South Asia. By applying a set of tools such as Combined Annotation Dependent Depletion (CADD), ANNOVAR, and Variant Effect Predictor (VEP), we highlighted 561 potentially detrimental variants from PJL data, and 7374 variants from ExAC South Asian data. Likewise, filtration from ClinVar for CVDs revealed 03 pathogenic and 02 likely pathogenic variants from PJL and 112 pathogenic and 42 likely pathogenic variants from ExAC South Asians. The comparison of derived allele frequencies (DAF) revealed many of these prioritized variants having two fold and higher DAF in Pakistani individuals than in other populations. The highest number of deleterious variants contributing to common CVDs in descending order includes hypertension, atherosclerosis, heart failure, aneurysm, and coronary heart disease, and for Mendelian and congenital CVDs cardiomyopathies, cardiac arrhythmias, and atrioventricular septal defects.

## Introduction

Cardiovascular diseases (CVDs) are the prime cause of death globally, accounting for over 31% of all the global deaths as estimated in 2012. The major proportion is endured by low- and middle-income countries, such as Pakistan [1]. The World Health Organization has reported 6.34 million disability adjusted life years (DALYs) due to CVDs in Pakistan in the duration 2000–2012, which was 19.6% of the burden by non-communicable diseases in the country [2]. The high prevalence and subsequent mortality attributed to CVDs is due to heritable and environmental contributing factors. The heritable component is polygenic and a result of complex interaction of many genes that confers an increased risk of CVD development [3].

Availability of population scale large DNA sequence datasets, such as 1000 Genomes Project [4] and the Exome Aggregation Consortium (ExAC) [5], have enabled researchers to explore variants frequencies of individual loci across populations and to highlight those related to local adaptations and disease susceptibility. The discovery of huge number of rare population or individual specific variants (MAF < 0.5%) in these genome sequencing projects is important for evaluating their contribution to the susceptibility and onset of diseases [6, 7]. Compared to the common variants, these rare variants more likely occur at evolutionary constrained site of proteins which have been kept conserved due to their functional importance. Such rare variants affect proteins composition in a more disruptive manner compromising or eliminating their function and affecting some phenotype [8]. The rate of emergence and distribution of such deleterious variants in populations is important in determining the patterns of underlying genetic load for diseases, because the increased accumulation of genetic load of diseases due to non-random segregation of deleterious variants is so detrimental that fixation or near-fixation of these mutations can play a significant role in the extinction of isolated populations with small effective population size [9, 10].

The effect of genetic variants for susceptibility or onset of diseases can be assessed in two ways using the DNA sequencing data: either screening the catalogued disease causing variants found already associated with certain disease by case-control studies, or prioritizing the detrimental variants, which have not been previously associated with diseases, by predicting their damaging effect [11]. The variant effect prediction tools make use of the available information such as the degree of conservation at the variant site and type of alteration in the protein composition, or its association with regulatory features and then predict the possible deleteriousness of variants under question [12]. As estimated earlier, on average a healthy person carries 281–515 missense substitutions, out of which 40–85 in homozygous state, predicted to be damaging and disease causing [11]. The presence of such deleterious variants in healthy individuals without showing apparent disease symptoms may be due to these variants being present in the heterozygous state, particularly for those that are associated with autosomal recessive disorders, having low penetrance, or being associated with a late disease onset. By genome wide association studies (GWAS), hundreds of common genetic variants have already been attributed to common CVDs such as hypertension, hypercholesterolemia, and coronary artery disease. Likewise genetic screenings have also identified many rare variants associated with Mendelian CVDs such as cardiomyopathies and arrhythmias. The common variants impart small cumulative risk in the onset of disease. The rare deleterious variants have been hypothesized to pose greater effect for these complex diseases [13]. Quantification of the mutational load for certain diseases provides a framework for understanding the overall effect of population-specific history on deleterious variation.

South Asia is one of the most densely populated regions having approximately one fourth of the world’s population [14]. This region faces severe socioeconomic inequities leading to serious health care issues [15]. Large scale ethnographic studies have shown that South Asians are at more risk to cardiovascular diseases than other ethnicities [16, 17]. CVDs account for 27% of the deaths in this region of the world, which is alarmingly high [18]. The age-standardized years of life lost due to CVDs has been increased in South Asia as compared to other regions. The incidence of acute myocardial infarction occurs about six years earlier than in western countries [19]. Likewise, the risk and prevalence of coronary artery disease is also considerably high in South Asians than in European populations [20].

Pakistan, the 2nd largest country of South Asia, and 6th largest country of the world (population 193.2 million) [14], is also facing serious health care issues. Estimates show that one in five adults of middle age may have sub-clinical coronary artery disease [21]. Prevalence of coronary artery disease in the local rural population has been reported to be 11.2% in one study [22]. Owing to the socio-demographic perspectives, consanguineous marriages are quite common in this region [23], which are possible cause of high prevalence of genetic disorders including cardiovascular diseases [24]. In this scenario, this study aims to estimate the underlying mutational burden of cardiovascular diseases in the Pakistani population. For this purpose, we make use of publically available genomic data of Pakistani population (Punjabi from Lahore; PJL) in the 1000 Genomes Project, and South Asians (SAS) in ExAC which predominantly contains samples from Pakistan as a cohort of the Pakistan Risk of Myocardial Infarction Study (PROMIS) [25]. For quantifying the mutational load, we applied two approaches, i.e. filtration of variants already reported to be associated with cardiovascular diseases in ClinVar database, and by predicting the functionally deleterious variants using variant effect prediction tools. In this analysis, we determined the concordance of mutational load of cardiac diseases between the two data sets, i.e., 1000 Genomes Project PJL, and ExAC SAS. We compared the allele frequencies of variants associated with these diseases to understand their relevance for estimating cardiovascular genetic risk in the Pakistani population in comparison with other continental populations.

## Methodology

### i. Preparation of genes lists

**Fig 1:**
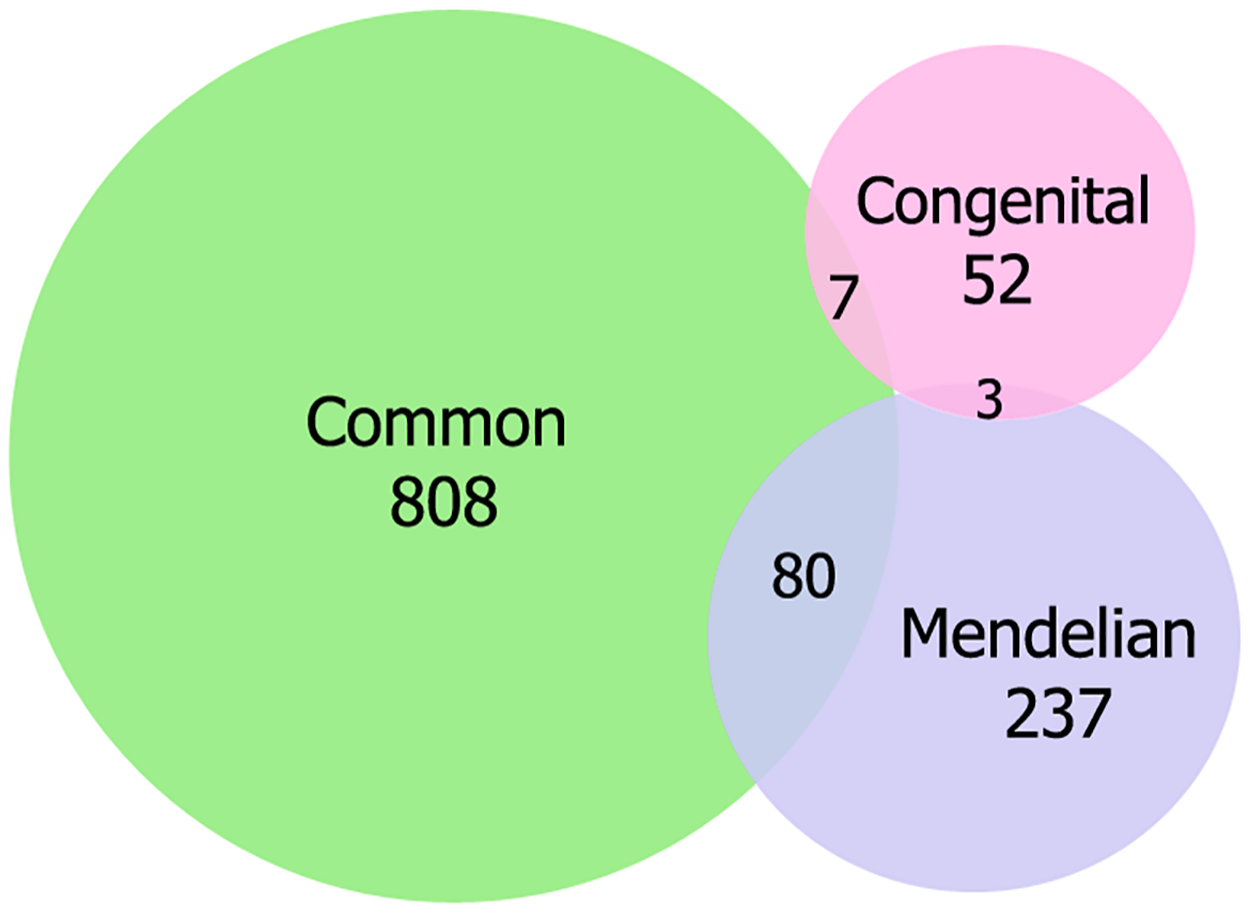
Venn diagram showing the number and overlap of genes associated with common, Mendelian and congenital cardiovascular diseases used in this analysis.

The genes reported to be associated with common, Mendelian, and congenital cardiovascular diseases were obtained primarily from three data bases, Online Mendelian Inheritance in Man (OMIM) [26], ClinVar [27], and Disease Ontology Annotation Framework (DOAF) [28]. The complete list of diseases at these databases were accessed and filtered for cardiovascular diseases using multiple terms related to CVDs such as ‘cardio’, ‘cardiac’, ‘heart’, ‘coronary’, ‘cardiomyopathy’, ‘myocardial’, ‘aneurysm’, ‘arteriopathy’, ‘atherosclerosis’, ‘septal defect’, ‘septal noncompaction’, ‘tetralogy of fallot’, ‘atrial’, ‘arterial’, ‘hypertension’, ‘QT syndrome’, ‘hypercholesterolemia’, ‘hyper triglyceridemia’ and some manually selected cardiac diseases. These terms were also compared with those in Human Phenotype Ontology [29] and WHO’s International Classification of Diseases (ICD-10) database. After manual curation through literature survey and refinement through GeneCards database [30], three lists comprising of genes relating to three categories of CVDs were prepared: one for common CVDs (n = 895 genes) such as hypertension, atherosclerosis, coronary heart disease, and heart failure, second for Mendelian CVDs (n = 320 genes) such as cardiomyopathies, cardiac arrhythmia, QT syndromes, and atrial fibrillation, and third for congenital CVDs (n = 62 genes) such as congenital heart disease, and atrioventricular septal defects. The lists of the selected genes associated with common, Mendelian and congenital CVDs are given in S1 Table. There was overlapping of few genes between these three categories of CVDs (Fig 1). The gene ontology terms to which these finally short listed genes belong were determined by UniProt Gene Ontology Annotation database for human version 2.0 [31] and plotted using the ‘BGI WEGO’ online Gene Ontology Tool [32].

### ii. Data set

Two data sets were used for estimating the mutational load of cardiovascular diseases in Pakistani population, i.e., the 1000 Genomes Project phase 3 data and ExAC release 0.3 data. The variants data for Pakistani population PJL (n = 96 individuals) was extracted from 1000 Genomes project data using the VCFtools [33]. This data of healthy persons was used for estimating mutational load of common, Mendelian and congenital CVDs. From ExAC database, the genetic variations related to South Asians (n = 8,276) were extracted and used as Pakistani data because it predominantly contained Pakistani individuals (n = 7,078) as part of the Pakistan Risk of Myocardial Infarction Study. This data was used for mutational load of Mendelian and congenital CVDs only because it already contained cohort of common cardiac diseases such as hypertension, hypercholesterolemia, and coronary artery disease apart from healthy controls [25].

### iii. Analysis pipeline

**Fig 2:**
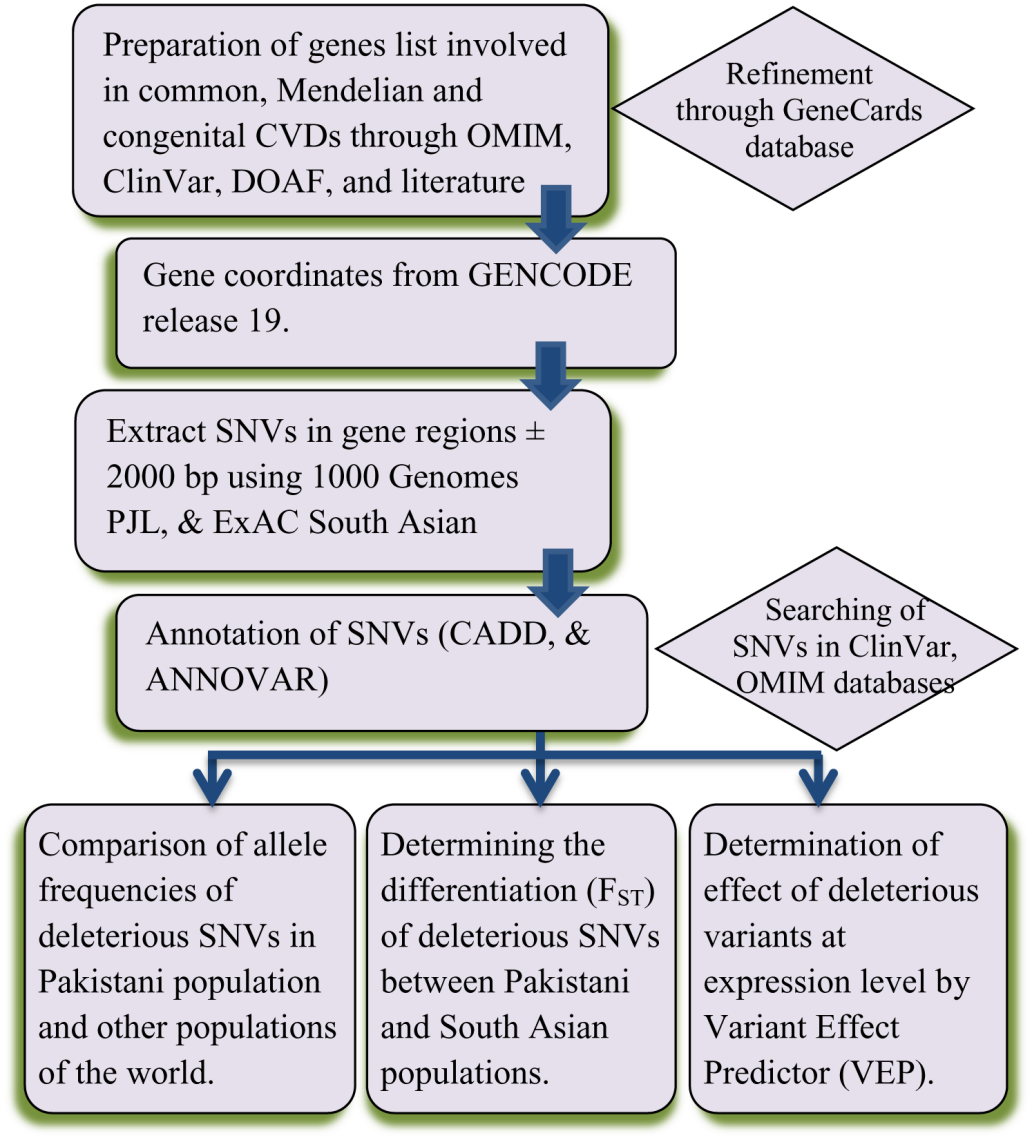
Analysis pipeline to determine the deleterious variants related to cardiovascular diseases in Pakistani population using 1000 Genomes Project PJL and ExAC SAS databases. (PJL = Punjabi in Lahore, Pakistan, ExAC SAS = South Asian in Exome Aggregation Consortium).

We developed a pipeline for computational analysis to determine the predicted deleterious effects of genetic variants based on functional annotations and assessing their prevalence using the common bioinformatics tools (Fig 2). The coordinates of the selected genes involved in cardiovascular diseases were obtained from GENCODE release 19 (gencode.v19.annotation.gtf), which is the final build of GENCODE mapped to the human GRCh37 reference assembly [34]. To cover the promoter regions of these genes in the analysis, 2000 was subtracted from the gene’s start position (the upstream region) and 2000 was added to the gene’s end position (the downstream position). In order to subset the variants of relevant genes bcftools-1.2.1 was used. For the current analysis, only the SNVs were used for prioritization. To determine the functional impact of the subset variants on proteins’ structure and function, three widely used tools were employed, i.e., the Combined Annotation Dependent Depletion (CADD) [35], PolyPhen-2 [36], and Sorting Intolerant from Tolerant (SIFT) [37]. These tools make use of machine learning approach to predict the effect of variants based on a number of factors including protein multiple sequence alignment, sequence- and structure-based features, and conservation across available homologous sequences [38]. Our approach was to prioritize missense (non-synonymous) variants preferably with low- and rare-allele frequency, because studies have shown that low- and rare-allele frequency variants are more in functional impact on proteins, whereby these are associated with complex phenotypes/disorders by changing the composition of proteins [39]. The annotation of the variants with CADD was performed using an in-house perl script (Supporting Information Script 1), while annotation with SIFT and PolyPhen-2 was performed with ANNOVAR [40]. We kept the criteria a bit stringent for filtration of harmful variants, such that an SNV was considered ‘functionally deleterious’ for which PolyPhen-2 HDIV score was > 0.957, SIFT score was < 0.05, and CADD phred-like score was 15 or higher (i.e. ≤ 1% percentile highest scores). We called such filtered SNVs as ‘predicted deleterious SNVs’ (dSNVs). The ancestral and derived states of deleterious variants were retrieved from online CADD annotation tool, which utilizes human-chimpanzee ancestral genome from the Ensembl EPO multiple alignments [41].

### iv. Comparison of the variants across the world populations

Population wise allele frequencies of predicted deleterious variants were retrieved by filter-based annotation with ANNOVAR using the 1000 Genomes and ExAC data frequency files. The comparison of allele frequencies for the two data sets (The 1000 Genomes and ExAC data) was carried out independently due to the difference in their data structure. The derived allele frequencies of predicted deleterious variants for cardiovascular diseases in Pakistani individuals of the 1000 Genomes Project were compared with all five major population groups i.e South Asian (SAS), European (EUR), Admixed American (AMR), African (AFR), East Asians (EAS), and Southeast Asian population ‘Malay’ [42]. Likewise, the derived allele frequencies of predicted deleterious variants in South Asian population of ExAC data were compared with all four populations of the same data i.e Non-Finnish Europeans (NFE), Latino (AMR), African/African American (AFR), and East Asian (EAS).

To find the populations wise genetic differentiation with respect to cardiovascular diseases, pair-wise Weir and Cockerham F_ST_ [43] values were calculated for the 1000 Genomes data, using the VCFtools. For this purpose, two approaches were employed, i.e F_ST_ calculation for all the genes which harbored the predicted deleterious SNVs in this analysis, and for deleterious SNVs only which were prioritized. Likewise, the relatedness of the populations based on the deleteriousness they harbored for cardiovascular diseases was assessed by Principal component analysis (PCA) using the PLINK tool (v1.90b3.30) [44] and verified by EIGENSOFT’s smartpca (version 3.0) [45].

### v. Searching the variants in ClinVar database

Annotation of the variants in genes set related to cardiovascular diseases were carried out using the ClinVar data release 20160104 [27]. The allele frequencies of ClinVar variants present in Pakistani individuals were retrieved by ANNOVAR annotation for both the 1000 Genomes populations and ExAC populations as described above. For comparison of allele frequencies among the populations, only those variants were selected with ClinVar significance ‘Pathogenic’, and ‘Likely_pathogenic’.

## Results

### i. Gene ontology

The grouping of genes under study according to their biological role was carried out using UniProt Gene Ontology Annotation database [31], which showed that most of the genes were primarily involved in binding, catalysis, and molecular transduction in a number of biological processes such as biological regulation, anatomical structure formation, cellular compartment organization and genesis, developmental process, metabolic process, and organismal process etc. (S1 Fig). Gene ontology shows that many genes are also related to structural processes of the heart representing the anatomical nature of cardiac diseases.

### ii. The mutational load of CVDs

**Fig 3:**
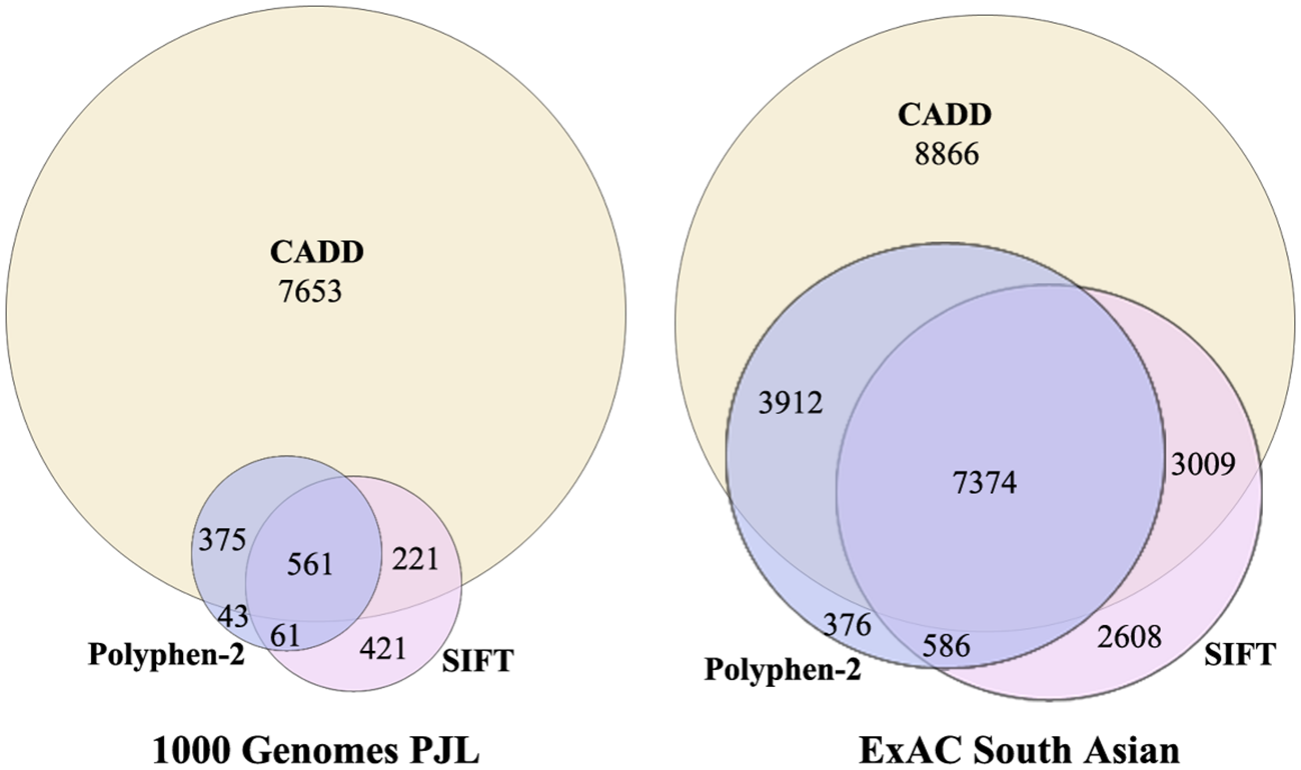
Venn-diagram indicating the number of SNVs predicted as deleterious by SIFT, Polyphen-2, and CADD in genes associated with cardiovascular diseases in this study. Considering the phred-like (scaled) score of 15 as deleterious, CADD predicts highest number of variants to be deleterious, due to the inclusion of non-coding variants.

All the SNVs in intronic, exonic, and flanking regulatory regions of our genes under study, as extracted from 1000 Genomes Project PJL and ExAC SAS data, were analyzed for mutational load by applying our analysis pipeline (Table 1). We calculated the proportions of synonymous, nonsynonymous, deleterious nonsynonymous, and homozygous deleterious SNVs from the two data sets. The proportions of nonsynonymous exonic SNVs (nonsynonymous SNVs/exonic SNVs), and deleterious nonsynonymous SNVs (deleterious nSNVs/nonsynonymous SNVs) was higher in ExAC SAS than in 1000 Genomes Project PJL (0.64 v.s. 0.51, and 0.26 v.s. 0.16 respectively). On the other hand, the proportion of synonymous SNVs and homozygous deleterious SNVs was observed to be higher in 1000 Genomes Project PJL than in ExAC SAS (0.45 v.s. 0.35, and 0.12 v.s. 0.04 respectively) (S2 Fig). After applying the prediction tools as described in analysis pipeline, 561 combinedly predicted deleterious SNVs were prioritized for common, Mendelian and congenital CVDs from 1000 Genomes Project PJL data, while there were 7374 combinedly predicted deleterious SNVs for Mendelian and congenital CVDs from the ExAC SAS data (Fig 3). Based on these findings from two data sets, the mutational load was observed to be higher for common CVDs than for Mendelian and congenital CVDs in Pakistani population. The highest number of deleterious variants contributing to common CVDs in descending order included hypertension, atherosclerosis, heart failure, aneurysm, and coronary heart disease, and for Mendelian and congenital CVDs cardiomyopathies (dilated and hypertrophic), cardiac arrhythmias, and atrioventricular septal defects.

**Table 1:** The number of variants subsetted from two datasets within the coordinates of our genes-sets of CVDs. The mutational load of deleterious SNVs per person was found to be higher for common CVDs than for Mendelian or congenital CVDs.

**Data Sets**	**1000 Genomes PJL**	**ExAC SAS**
Sample size	96	8276
CVDs related genes analyzed here	1187	379
Subset of variants in these genes	363543	71816
Exonic variants	6941	44357
Upstream variants	4668	80
Downstream variants	4752	09
5’-UTR	1573	1075
3’-UTR	7541	1694
**Predicted Consequences of Variants:**		
Non-synonymous SNVs	3521	28305
‘Combinedly predicted deleterious’ with SIFT, Polyphen-2, and CADD phred score 15 (dSNVs)	561	7374
Homozygous dSNVs	69	306
Loss of Function (LoF) variants in dSNVs	05	142
Per person dSNVs	5.84	0.89

### iii. Filtration of variants in ClinVar

**Fig 4:**
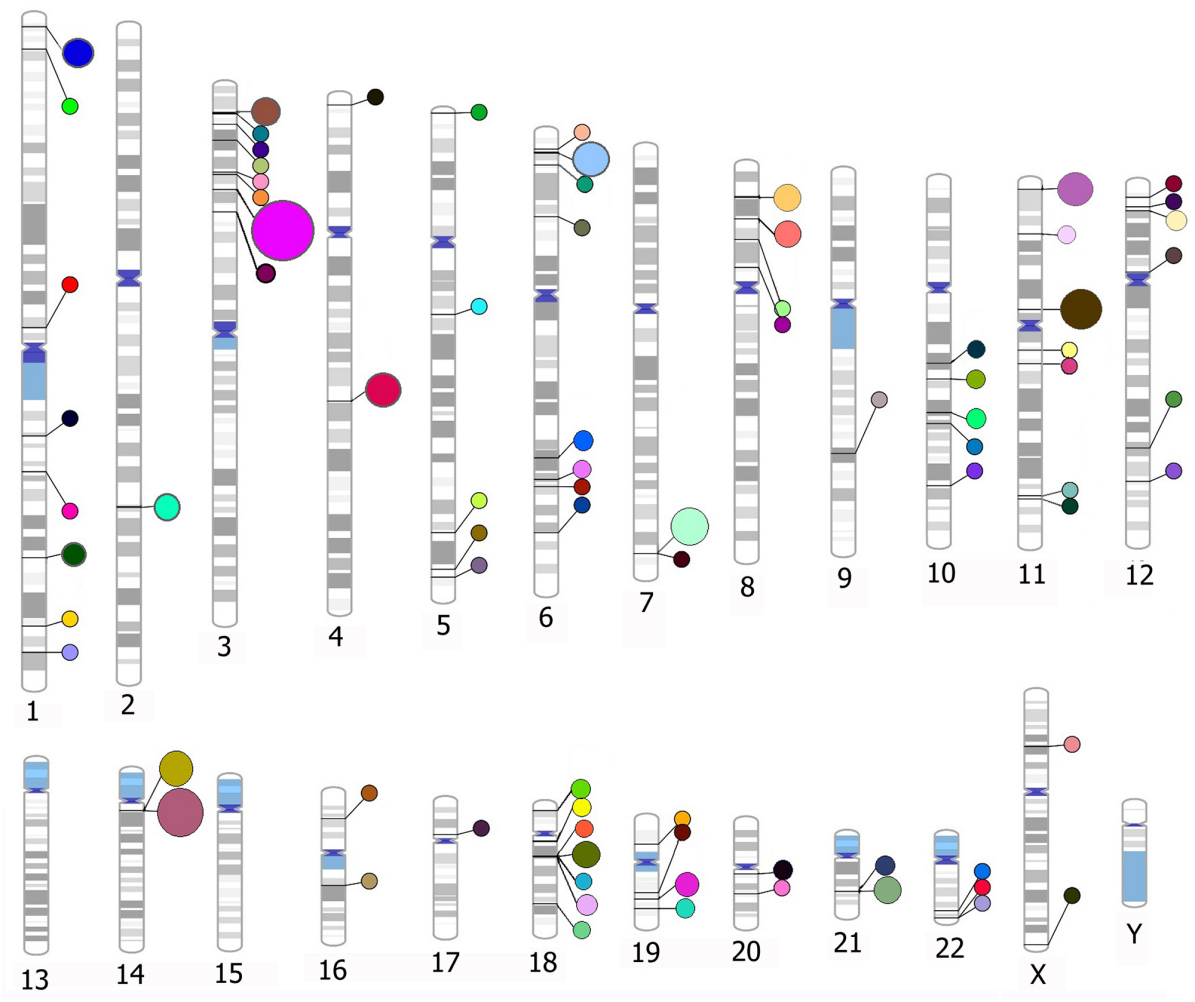
Genomic positions of genes harboring the variants associated with CVDs as filtered from the ClinVar database. One colour of circles beside the chromosomes denotes one gene. The size of circle corresponds to the number of associated variants in that gene. The loci on chromosomes 3, 11, 14, & 18 are richer in variants with clinical significance for CVDs.

The filtration of our set of variants based on pathogenicity in ClinVar database identified several variants associated with Mendelian and congenital cardiovascular disorders. There were 03 variants with ClinVar significance ‘Pathogenic’, and 02 variants with ‘likely Pathogenic’, significance for CVDs in 1000 Genomes Project PJL population (S2 Table, sheet A). The three pathogenic SNVs (rs201654872, rs115372595, and rs201680145) contribute to dilated cardiomyopathy, atrioventricular septal defect, and cerebral autosomal dominant arteriopathy respectively. The annotation with online VEP tool showed that two pathogenic missense SNVs rs201654872 [Val/Met] and rs201680145 [Arg/Cys] are linked with CCCTC-binding factor site (CTCF_binding_site). The CTCF_binding_sites are major determinants of long-range interactions (looping) of chromatins which alter gene expression [46]. The third pathogenic missense SNV rs115372595 [Ala/Val] is also linked with regulatory region (open chromatin region). The open chromatin sites tend to be near the transcription start site and play a role in gene expression coincident with CTCF binding sites [47]. The two ‘Likely Pathogenic’ variants (rs193922669, and rs77613865) contribute to arrhythmogenic right ventricular cardiomyopathy and hypertrophic cardiomyopathy respectively. The missense SNV rs193922669 causes Arg/His substitution in desmoplakin protein, while rs77613865 is a splice region variant, and is also linked with open chromatin region affecting the expression of myomesin 1 (*MYOM1)*. On the other hand, in ExAC South Asian data, 112 ‘Pathogenic’ SNVs, 42 ‘Likely Pathogenic’ SNVs were filtered (S2 Table, sheet B). As a whole, 73 (47.40%) of the filtered SNVs belonged to various forms of cardiomyopathies, 38 (26.68%) were related to Long_QT syndrome, and 8 (5.19%) to different forms of atrioventicular defects. It was also noted that 31 SNVs had multiple significances for more than one type of Mendelian or congenital CVDs. The allele frequencies of filtered variants were compared which highlighted 11 variants having allele frequency significantly higher in SAS than in other populations (Table 2). Functional consequences with online VEP tool showed 13 variants with Loss of Function (LoF) effect, and 23 regulatory region variants (S2 Table, sheet B). We highlighted the genomic locations of genes harboring the ClinVar variants associated with common, Mendelian and congenital CVDs in Pakistani population (Fig 4). The loci of different genes such as *SCN5A* on chromosome 3, *KCNQ1* and *MYBPC3* on chromosome 11, *MYH6* and *MYH7* on chromosome 14, and *KCNE1* and *KCNE2* on chromosome 21 were found enriched for clinically significant variants.

**Table 2:** ClinVar’s Pathogenic and Likely pathogenic variants associated with CVDs having significantly higher percent allele frequency in SAS than in other populations.

CHR	POS	ID	REF	ALT	Gene	Clinical Significance	ExAC_AF	AFR_AF	AMR_AF	EAS_AF	FIN_AF	NFE_AF	SAS_AF	Disease
1	3329208	rs397514743	A	G	PRDM16	Pathogenic	0.0264	0	0	0.0375	0	0	0.3118	Left_ventricular_noncompaction_8
1	3347452	rs201654872	G	A	PRDM16	Pathogenic	0.3529	0.0103	0.0087	0.0116	0	0.0030	2.5630	Dilated_cardiomyopathy_1LL
3	38592408	rs137854619	C	T	SCN5A	Pathogenic	0.0305	0	0	0	0	0.0060	0.1999	Long_QT_syndrome_2/3| Congenital_long_QT_syndrome
3	38622640	rs199473183	A	G	SCN5A	Pathogenic	0.0282	0	0	0	0	0	0.2183	Congenital_long_QT_syndrome
3	46899901	rs145520567	C	T	MYL3	Likely Pathogenic	0.0264	0	0.0086	0	0	0.0090	0.1514	Cardiomyopathy
6	7542236	rs121912998	G	A	DSP	Pathogenic	0.1608	0.0542	0.1018	0	0.0773	0.3291	0.5701	Arrhythmogenic_right_ventricular_ cardiomyopathy\x2c_type_8
6	7583050	rs193922669	G	A	DSP	Likely Pathogenic	0.0239	0	0	0	0	0.0045	0.1575	Arrhythmogenic_right_ventricular_ cardiomyopathy
8	11615928	rs56208331	G	A	GATA4	Pathogenic	0.2117	0	0.0173	0	0.0151	0.0375	1.3687	Atrial_septal_defect_2| Tetralogy_of_Fallot
11	47354209	rs199669878	C	T	MYBPC3	Likely Pathogenic	0.0405	0.0118	0.0306	0	0	0.0372	0.1447	Cardiomyopathy
14	23894554	rs376754645	C	T	MYH7	Likely Pathogenic	0.0222	0	0.0086	0.0231	0	0.0045	0.1272	Familial_hypertrophic_cardiomyopathy_1
18	3149140	rs77613865	T	G	MYOM1	Likely Pathogenic	0.3879	1.616	0.0866	0	0	0.0090	1.7470	Hypertrophic_cardiomyopathy

### iv. Comparison of derived allele frequencies of predicted deleterious variants across continental populations

**Fig 5:**
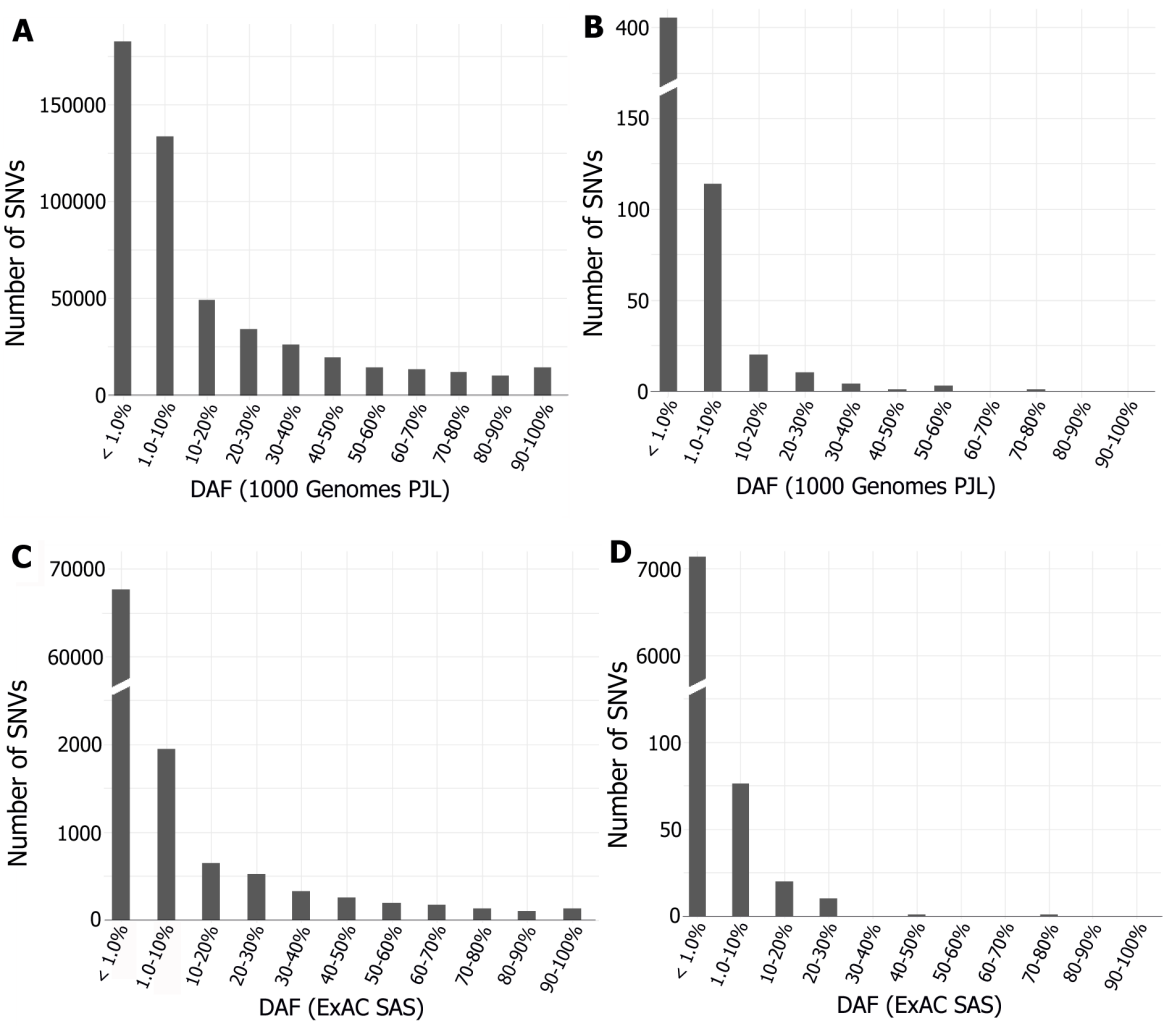
Allele frequency spectrum (AFS) of all and deleterious SNVs in genes related to CVDs. **A.** All SNVs in 1000 Genomes PJL, **B.** Deleterious SNVs in 1000 Genomes PJL. **C.** All SNVs in ExAC SAS, **D.** Deleterious SNVs in ExAC SAS. There are more deleterious SNVs in common DAF bins (>5%) in 1000 Genomes PJL than in ExAC SAS.

**Fig 6:**
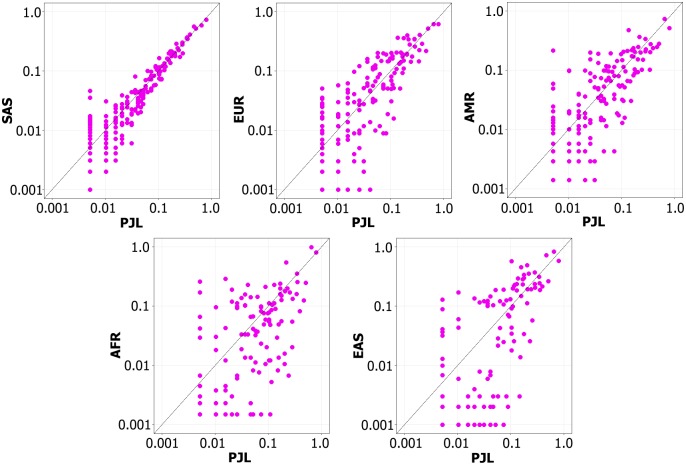
Based on the shared predicted deleterious SNVs, the comparative distribution of allele frequencies in Pakistani population versus all five groups of 1000 Genomes Project. The variants at diagonal line have almost equal DAF in both the populations being compared, whereas, the variants to the right of diagonal line have higher DAF in PJL, and variants to the left of diagonal line have higher DAF in the other population being compared.

Derived allele frequency spectrum of all the SNVs and deleterious SNVs in our genes-set of CVDs filtered from 1000 Genomes Project PJL data and ExAC South Asian data, revealed that majority of the deleterious variants were of rare allele frequency. The proportion of common allele frequency deleterious SNVs (AF > 5%) was found to be 11.59% in 1000 Genomes Project PJL for common, Mendelian and congenital CVDs, while it was found only 00.62% for Mendelian and congenital CVDs from ExAC SAS (Fig 5). The comparison of derived allele frequencies of predicted deleterious SNVs was carried out with other major population groups within their respective data set. This comparison revealed two important findings: (a) The extent of private and shared deleterious SNVs between the Pakistanis and other populations, and (b) the number of deleterious SNVs with higher derived allele frequency in the Pakistani population (or in South Asian in case of ExAC data) than in other populations. It was noted that the extent of sharing deleterious SNVs was different with different populations groups. Overall, 33.16% of the predicted deleterious SNVs were private to PJL in 1000 Genomes Project data, the derived allele frequencies of which varied from 0.0052 to 0.0260, while 66.84% SNVs were shared with derived allele frequencies ranging from 0.0052 to 0.7968. So, it was evident that among the predicted deleterious SNVs, the private proportion contained only rare variants (DAF < 0.5%), while the shared proportion contained both rare (47.50%) and common variants (52.50%) within this category. On the other hand from ExAC data analysis, greater proportion of deleterious SNVs (i.e. 56.64%) was private to SAS, while 43.36% deleterious SNVs were shared with other populations. Among the shared deleterious SNVs, the proportions of those having higher derived allele frequencies in Pakistani population were found greater in all five comparisons conducted within 1000 Genomes Project populations. Whereas, for ExAC data, the proportion of shared deleterious SNVs with higher derived allele frequency in SAS was greater than Non-Finnish European only (Table 3). Interestingly it was noted that the proportion of shared deleterious SNVs of Mendelian and congenital CVDs with other populations (ExAC data) was less than the proportion of shared deleterious SNVs of common, Mendelian and congenital CVDs (1000 Genomes Project data) except for the comparison with the European population (S3 Fig). This comparison also revealed that there was comparatively less difference in derived allele frequencies of most of the deleterious SNVs between 1000 Genomes Project PJL and rest of 1000 Genomes Project South Asian populations, however, in some cases a significant difference up to 5.2 times higher was observed. The maximum difference of derived allele frequency of shared deleterious SNVs with Americans was 22.32 times higher in PJL, for Europeans 41.67 times higher in PJL, whereas, great frequency difference was observed with Africans and East Asians where the maximum derived allele frequency difference was calculated to be 72.19 times higher in PJL (Fig 6) (S3 Table, sheet A). The median DAF was found to be higher in PJL as compared with SAS, EAS, AMR, and AFR populations, while it was lower in PJL when compared with EUR populations (S4 Fig). Likewise, for comparisons of derived allele frequency of ExAC SAS, the highest difference was observed with NFE i.e. 1098 timers higher in SAS. The maximum difference for other ExAC populations was 858 times than EAS, 347 times than AFR, 290 times than AMR, and 64 times than FIN populations (S3 Table, sheet B).

**Table 3:** The proportion of shared deleterious SNVs (sdSNVs) with other populations of 1000 Genomes Project and ExAC. The proportion of sdSNVs with a higher DAF in PJL was greater in all pairwise population comparisons. On the other hand, from the comparison of ExAC_SAS with other populations, the proportion of sdSNVs with higher DAF in SAS was greater than NFE (Non-Finnish Europeans) only, while, it was less than AMR (Americans), AFR (Africans), EAS (East Asians), and FIN (Finnish) populations.

**1000 Genomes PJL**	**Total dSNVs**	**Private dSNVs**	**deleterious SNVs shared with different populations**		**Proportion (shared with pop/total shared dSNVs)**	**SNVs with higher DAF in PJL**	**SNVs with lower DAF in PJL**	**Proportion (higher DAF SNVs/shared in pop)**
	561	185	shared with SAS	376	1.000	282	94	0.750
			shared with EUR	199	0.529	108	91	0.543
			shared with AMR	171	0.455	99	72	0.579
			shared with AFR	157	0.418	119	38	0.758
			shared with EAS	127	0.338	84	43	0.661
**ExAC SAS**	**Total dSNVs**	**Private dSNVs**	**deleterious SNVs shared with different populations**		**Proportion (shared with pop/total shared dSNVs)**	**SNVs with higher DAF in SAS**	**SNVs with lower DAF in SAS**	**Proportion (higher DAF SNVs/shared in pop)**
	7374	4170	shared with NFE	2480	0.774	1883	597	0.759
			shared with AMR	1211	0.378	473	738	0.391
			shared with AFR	1202	0.375	445	757	0.370
			shared with EAS	893	0.279	268	625	0.300
			shared with FIN	478	0.149	123	355	0.257

### v. Functional consequences of deleterious variants

We then grouped the deleterious variants of both data sets according to their functional consequences to point out LoF variants, including ‘stop_gained’, ‘stop_lost’, ‘start_lost’, ‘frameshift change’, and ‘splice donor or acceptor variants’ which possess the most damaging effect to proteins structure and function [48], by using the online Variant Effect Predictor tool [49]. The analysis divulged 03 LoF variants in 1000 Genomes Project PJL individuals, out of which 2 were in homozygous state. These include homozygous ‘rs2228570’ (start lost), heterozygous ‘rs371316552’ (stop gain), and homozygous ‘rs117054298’ (splice acceptor variant). The derived allele frequency of ‘rs2228570’ was found to be quite high in the PJL population i.e. 79.68%. Comparison of derived allele frequency of this variant with global populations showed that this is prevalent in almost all populations with higher frequencies ranging from 51.73% in Americans to 81.09% in Africans. This variant lies within vitamin D receptor (*VDR*), whose 6 out of 9 transcripts were found to be affected with LoF mutation, and is associated with many disease conditions including the hypertension [50, 51]. The ‘rs371316552’ SNP belongs to cathepsin B (*CTSB*), whose increased expression has been reported to pose a risk for atherosclerosis and myocardial infarction in rat models [52]. The third LoF SNP ‘rs117054298’ belongs to insulin-like growth factor (IGF) binding protein-1 (*IGFBP1*), whose splice site of one transcript ENST00000457280 is disrupted and contributes to atherosclerosis [53]. Likewise, 30 LoF variants were found in ExAC South Asians, out of which 2 were in homozygous state (S4 Table).

### vi. Differentiation of deleterious variants

**Fig 7:**
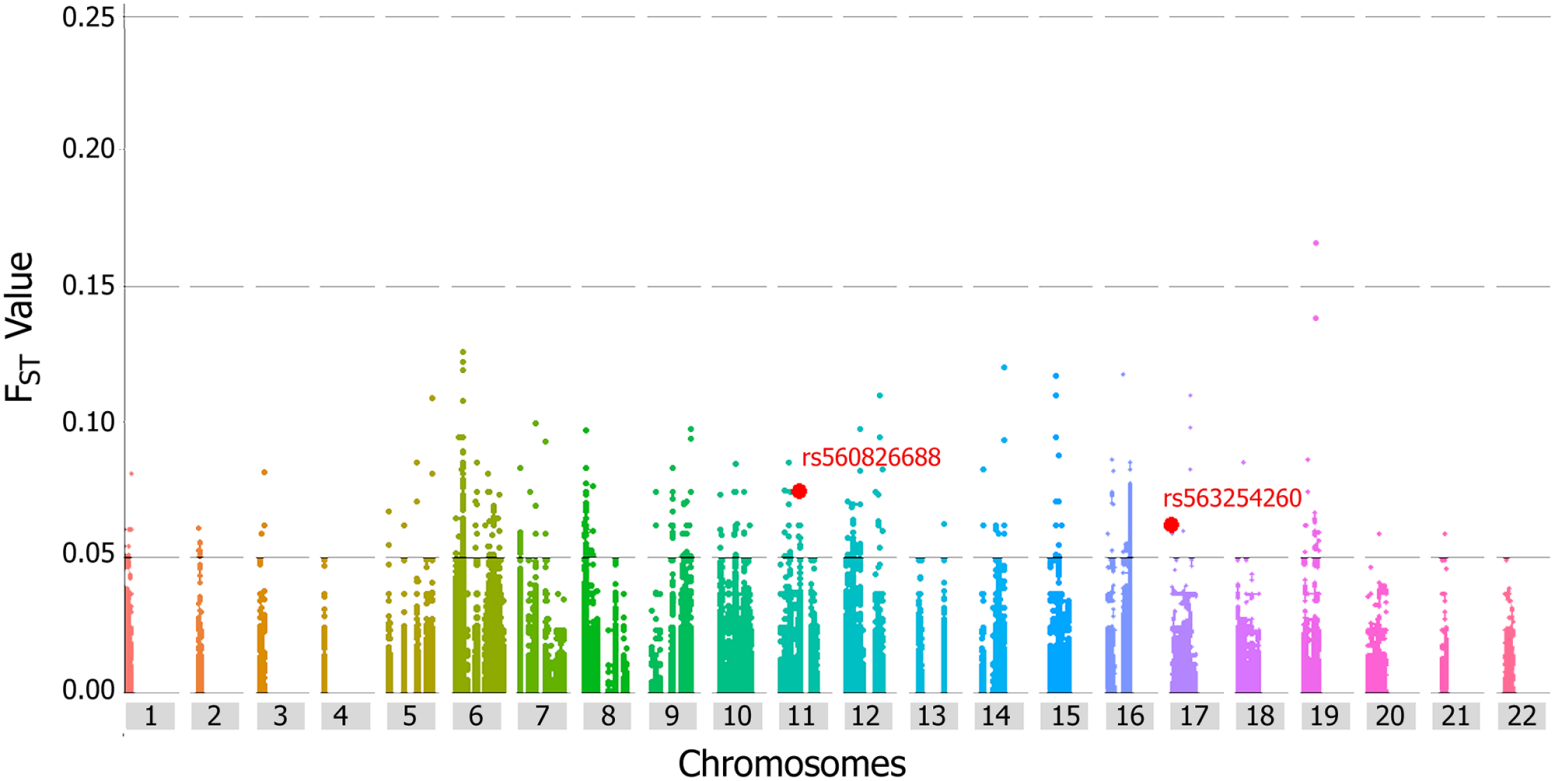
Manhattan plot for pair wise F_ST_ values between the PJL and other South Asian populations of 1000 Genomes Project. The plot is for selected genes which harbored the deleterious SNVs for cardiovascular diseases, as filtered in this analysis. The horizontal lines at 0.05, and 0.15 positions of y-axis represent boundaries of less differentiated (F_ST_ <0.05), moderately differentiated (F_ST_ 0.05–0.15) and highly differentiated (F_ST_ 0.15–0.25). The two deleterious SNVs, which are moderately differentiated in PJL, are highlighted red.

Data from whole genome/exome sequencing projects can be used to find out the extent of differentiation among populations based on the differences in allele frequencies of nonsynonymous variants. The presence of variants with highly differentiated frequencies among the populations provides a direction to fine-map signals of local adaptation as well as susceptibility to diseases [54]. In this study, the differentiation was determined by calculating the Weir and Cockerham F_ST_ in two ways: (1) F_ST_ calculation for PJL versus other South Asian (SAS) populations of 1000 Genomes Project using all the SNVs in genes harboring the filtered deleterious SNVs for cardiovascular diseases, and (2) F_ST_ calculation for PJL versus all other populations in 1000 Genomes Project. The F_ST_ calculated for PJL versus SAS populations showed mean F_ST_ value of 0.00134, while the mean F_ST_ for deleterious SNVs was calculated as 0.00638, which is 4.76 times higher than the mean F_ST_ of all SNVs. Two deleterious SNVs (rs560826688 and rs563254260) were found moderately differentiated (F_ST_ value 0.05–0.15) from other South Asian populations ranking well above top 1% within all SNVs i.e., at top 0.11% and 0.29% respectively (Fig 7). The derived allele frequency of rs560826688 is 0.031, and belongs to *LRP5* involved in hypertension [55], and derived allele frequency of rs563254260 is 0.026 and lies in *SERPINF1* which relates to obesity and hypertension [56]. In addition to these, one highly differentiated (F_ST_ value 0.15–0.25) SNV rs539962979 with F_ST_ value 0.16597 was also observed in *DMPK* which has been reported to be involved in cardiomyopathy [57]. Likewise, the F-statistics performed for PJL versus all other populations of 1000 Genomes Project, showed comparatively higher differentiation than with the SAS populations, where the mean F_ST_ of 0.0031 for all SNVs, and 0.0392 for deleterious SNVs was calculated. The proportions of moderately, highly, and severely differentiated SNVs was calculated within the pools of deleterious SNVs and all SNVs separately. This comparison showed that deleterious pool had higher proportion of moderately differentiated SNVs (S5 Fig). Besides this, 08 highly differentiated and 02 severely differentiated deleterious SNVs were also observed (S5 Table).

**Fig 8:**
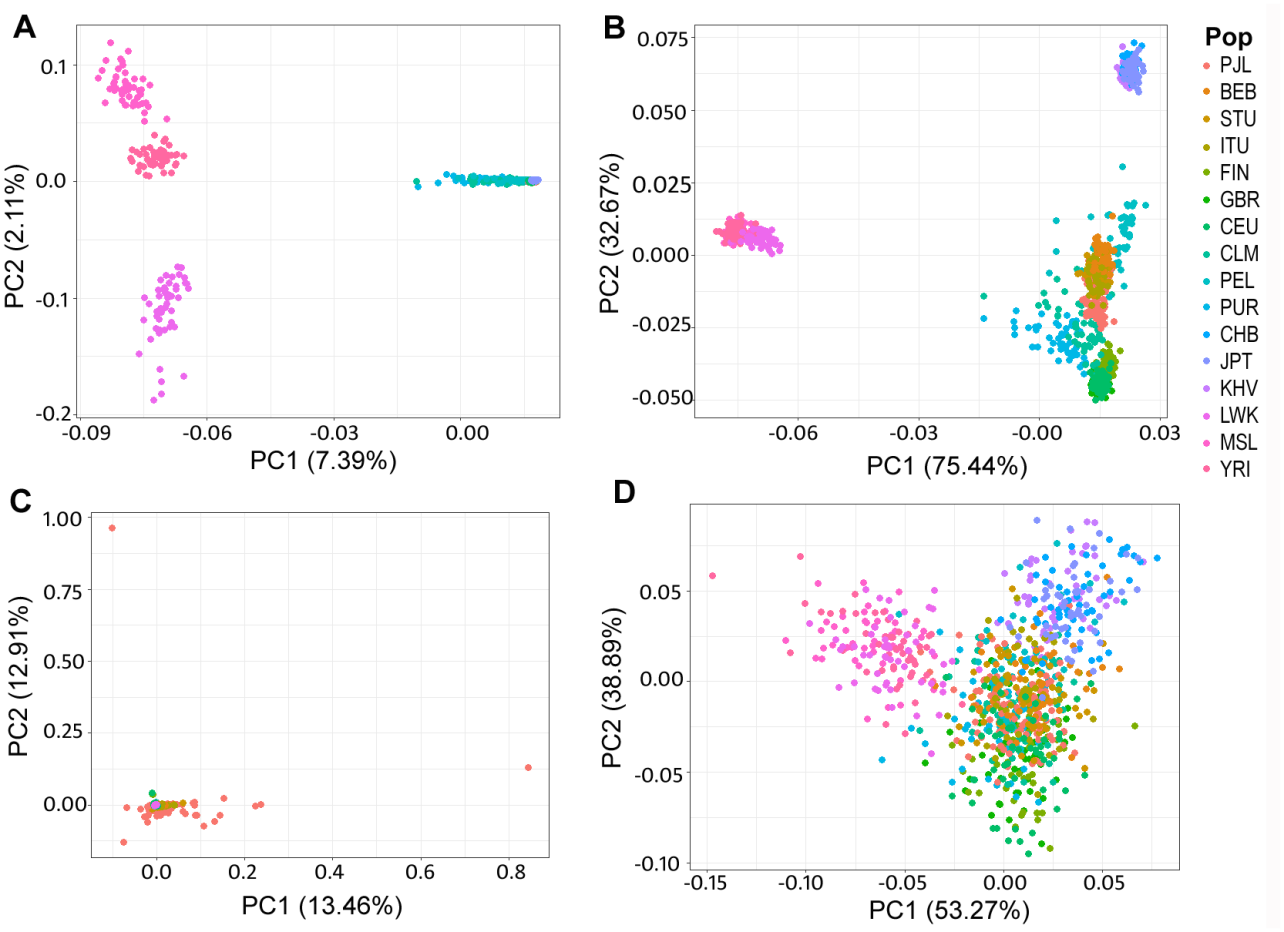
Principal Components Analysis (PCA) based on genes involved in CVDs. **A.** PCA based on all variants in our genes set with AF≤5.0%, **B.** PCA based on all variants in our genes set with AF > 5.0%. **C.** PCA based on deleterious variants with AF ≤ 5.0%, **D.** PCA based on deleterious variants with AF > 5.0%. PJL appears to be diverging out from other populations of the world based on low and rare allele frequency deleterious variants (AF≤5.0%) (Panel C) when compared to the analysis with all low and rare allele frequency variants in our genes-set (Panel A). For common allele frequency variants (AF>5.0%), populations appeared in three distinct groups based on all variants (Panel B), while they appeared to be merging when analyzed with deleterious variants only (Panel D).

The observed difference in allele frequencies and calculated F_ST_ values of functionally predicted deleterious SNVs between PJL and rest of the global populations gave a clue for stratification of the world populations based on mutational burden for cardiovascular diseases. So, principal component analysis was performed for the deleterious SNVs and all the SNVs of our genes-set from 1000 Genomes Project data. The analysis with all the low and rare allele frequency SNVs of our genes-set (DAF ≤ 5.0%) showed all the populations grouped together while African populations making distinct group (Fig 8A). The analysis with low and rare deleterious SNVs showed all populations grouped at one place while PJL scattering from them (Fig 8C). Likewise, the PCA with all common allele frequency SNVs (DAF > 5.0%) of our genes-set suggested three distinct groups of world populations in which South Asian, European, and American populations appeared as one group. The African populations and East Asian populations grouped separately in this analysis (Fig 8B). In the PCA with deleterious common allele frequency SNVs, the afore-mentioned groups appeared to be merging together (Fig 8D).

**Fig 9:**
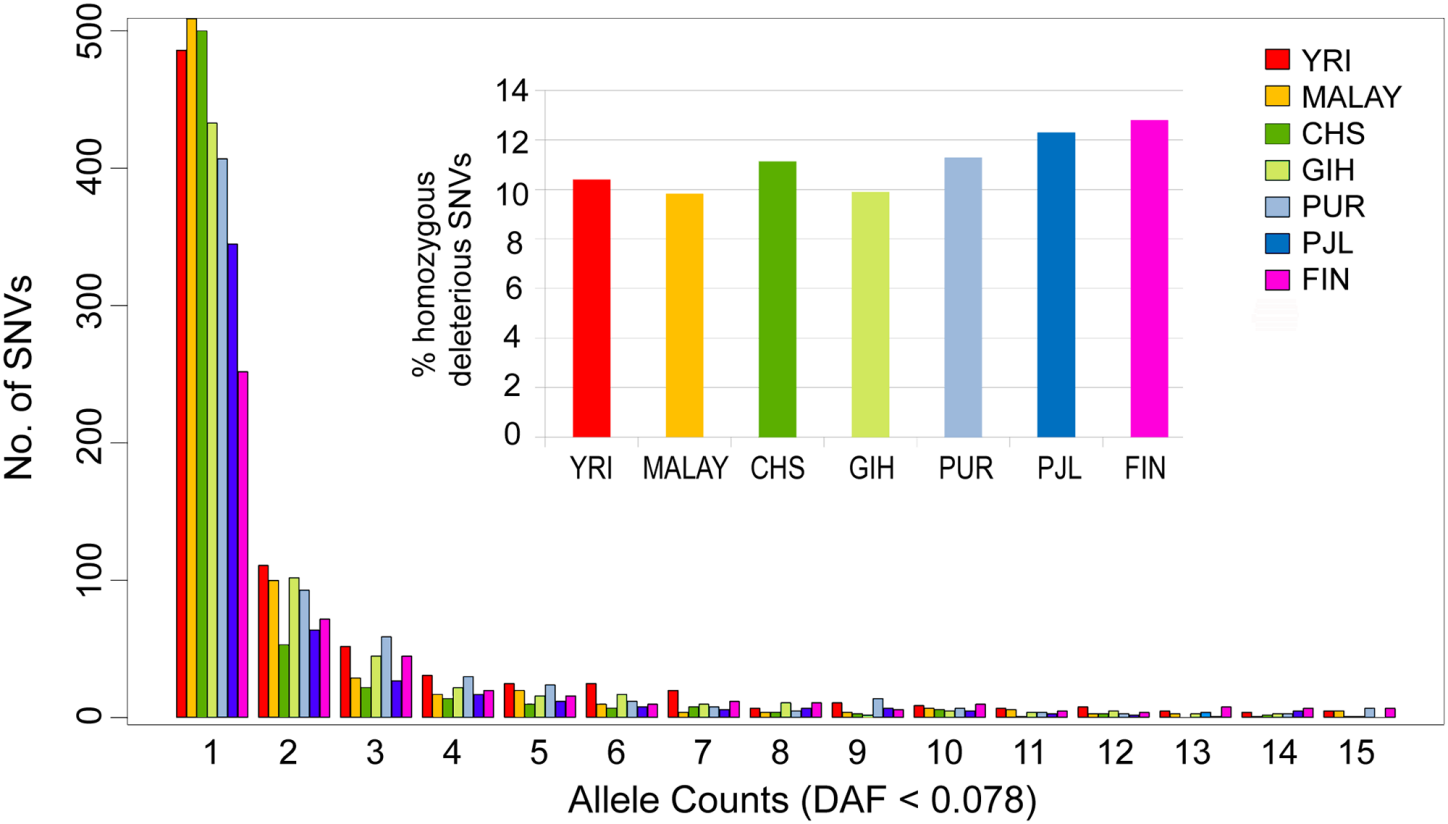
Comparison of site frequency spectrum for PJL, 5 other populations of 1000 Genomes Project, and one Southeast Asian population ‘Malay’, using the data of same number of individuals (n = 96) of each population for normalization. **A.** Comparison of low frequency deleterious SNVs in genes set of cardiovascular diseases. **B.** Percent homozyous deleterious SNVs in each population.

Using the same set of genes, the burden of common and Mendelian, and congenital cardiovascular diseases was also determined for one population from each of five major population groups of 1000 Genomes Project i.e., Yoruba in Ibadan (YRI) in Africa, Southern Han Chinese (CHS) from East Asian, Gujarati Indian from Houston (GIH) in South Asia, Puerto Ricans (PUR) from America, Finnish (FIN) in Finland, and Malay of East Asia which is not part of 1000 Genomes Project. This empirical estimation showed excess of deleterious derived rare variants (singletons) in YRI and Malay populations, while FIN and PJL populations harbored the least number of deleterious derived singletons (Fig 9A). Furthermore, the proportion of homozygous deleterious derived SNVs was observed to be second highest in PJL after the Finnish population (PJL 12.30%, Finnish 12.79%, Fig 9B).

## Discussion

In this study, we have quantified the mutational burden for common, Mendelian, and congenital cardiovascular diseases in Pakistani population and compared it with other populations of the world. This quantification of mutational load by assessing the functionally deleterious SNVs gave a clue for high prevalence of common CVDs in this region [58]. The observed higher mutational load for common CVDs than for Mendelian and congenital CVDs can be explained that common CVDs are polygenic where large number of deleterious variants with modest-to-weak effect contribute to them, whereas Mendelian CVDs are monogenic or oligo-genic where few rare variants pose greater effect in the phenotype [6]. These modest-to-weak effect deleterious variants spread in the populations and raised in allele frequencies along with neutral variants during the rapid population expansion [59]. However, the allele frequencies of deleterious genetic variants contributing to certain human diseases may be different among populations, according to their historical modes of expansion, role of evolutionary forces, and bottlenecks. Highly deleterious variants are purged by purifying selection from the population and are rare [6, 58, 60]. So, more deleterious variants were observed in common DAF bins (>5%) for 1000 Genomes Project PJL than for ExAC SAS (Fig 5). This was further evaluated by calculating the proportions of rare-, low- and common-DAF deleterious SNVs for both data sets. The proportion of common-DAF deleterious variants was found to be 11.03% in 1000 Genomes PJL, while it was only 0.54% in ExAC SAS (S6 Fig). The higher proportion of common-DAF deleterious SNVs in 1000 Genomes Project PJL can also be explained by previous findings that the variants with very small detrimental effect for complex disorders can survive in populations for thousands of years without undergone purifying selection [61], or these contribute to late onset of diseases. Further, the genes contributing to Mendelian disorders are being under tight selection, while those contributing to complex disorders show interplay of negative and positive selection due to some balancing effect [62]. For example, the high frequency deleterious SNV rs2228570 (start lost of *VDR*) has been reported to contribute to hypertension [50, 51], and also protects from intervertebral disc degeneration [63]. This comparison also revealed that the proportion of rare-DAF SNVs was higher in deleterious pool than in total SNVs pool for both the data sets (S6 Fig). The comparatively higher proportion of rare SNVs in deleterious pool (62.80% in 1000 Genomes Project PJL and 98.63% in ExAC SAS) is consistent with earlier studies [64], and can be inferred in the light of population demography i.e., the genes involved in cardiovascular diseases have acquired such rare deleterious SNVs in the Pakistani population because of rapid population expansion in recent times [65]. The effect of neutral forces is further strengthened by the larger proportion of private deleterious SNVs (Table 3), because the most recently emerged SNVs also tend to be private in a population, and those population specific rare variants are even more likely to be deleterious for certain diseases [66].

The presence of ClinVar’s pathogenic and likely pathogenic variants of CVDs in 1000 Genomes Project PJL and ExAC SAS also represents the underlying burden of these diseases in Pakistani population. The variants filtered in PJL and SAS were found to be associated with Mendelian and congenital CVDs only. The major proportion of filtered variants was related to cardiomyopathies (47.8%), long_QT syndrome (23.9%), cardiac arrhythmia (8.8%), and atrio-ventricular septal defects (5.0%). Among the 11 SNVs with higher allele frequency in SAS than in other populations (Table 2), 8 were related to cardiomyopathies. In addition to SNVs, we filtered a 25-bp deletion (rs36212066) in intron 32 of *MYBPC3* (cardiac myosin binding protein C), which was reported to be related with cardiomyopathies and present in populations of Indian origin with MAF ~4% [67]. In this analysis, this deletion was found with MAF 3.1% in both the 1000 Genomes PJL, and ExAC SAS. In PJL, it was present in heterozygous form, while in ExAC SAS, 11 were in homozygous state and 495 in heterozygous state.

Owing to the current understanding that genetic burden of common diseases may be different for populations according to their past histories [58], we hypothesized that deleterious variants imparting their role in cardiovascular diseases in Pakistani population may had differentiated from South Asian populations in a more recent time. But our results from the pair-wise calculated F_ST_ values were persistent with previous findings that variants contributing to common diseases are not well differentiated [68]. The two deleterious SNVs, rs560826688 (DAF 0.0312) and rs563254260 (DAF 0.0260) which are moderately differentiated from other South Asians, are also severely differentiated form all populations of 1000 Genomes Project. These correspond to *LRP5* (which encodes Low Density Lipoprotein Receptor-Related Protein 5) and *SERPINF1* (which encodes Pigment Epithelium Derived Factor (PEDF) belonging to Serpin Peptidase Inhibitors superfamily) respectively; and both contribute to hypertension [55, 56]. Their evolution to comparatively higher frequencies in Pakistani population may be due to genetic drift having some bona fide effect masking their role in hypertension in this region [69]. The severely and highly differentiated SNVs from all 1000 Genomes Project populations (S5 Table) is also in accordance with the calculated higher burden of CVDs in Pakistan i.e., hypertension, atherosclerosis, heart failure, cardiomyopathy, and septal defects. Overall, comparatively less differentiation of deleterious SNVs was observed from South Asian, European and American populations (S5 Fig) representing the less evolution of genetic factors responsible for the susceptibility of cardiovascular diseases, while the observed high differentiation with African and East Asian populations represents their diversity or differential susceptibility to cardiac diseases, which is persistent with the influence of geography, language and ethnicity on genetic variation in those regions [70]. The PJL was also found grouped together with other South Asians, Europeans and Americans based on the genetics of cardiovascular diseases as carried out in this analysis (Fig 8). This paradigm also correlates with the route of expansion of modern humans after the migration from Africa. In future, the prioritized variants can be assessed and validated empirically by DNA sequencing of these genes in large cohort of relent cardiac patients.

## Web resources

The URLs for data used and tools presented herein are:

International Classification of Diseases: http://apps.who.int/classifications/icd10/browse/2010/en

1000 Genomes Project phase 3 data: ftp://ftp.1000genomes.ebi.ac.uk/vol1/ftp/release/20130502/

ExAC release 0.3 data: ftp://ftp.broadinstitute.org/pub/ExAC_release/release0.3/

UniProt Gene ontology annotations dataset: ftp://ftp.ebi.ac.uk/pub/databases/GO/goa/HUMAN/

Gene Ontology Tool: http://wego.genomics.org.cn/cgi-bin/wego/index.pl

bcftools-1.2.1 http://www.htslib.org/download/

Online CADD annotation tool: http://cadd.gs.washington.edu/score

Online VEP tool: http://asia.ensembl.org/Tools/VEP

## Supporting information

S1 Script Perl script to annotate the variants with CADD scores. (PL)

S1 Fig Categorization of genes involved in CVDs based on cellular, molecular, and biological processes. (TIF)

S2 Fig Proportions of synonymous SNVs, nonsynonymous SNVs, deleterious non-synonymous SNVs (as determined by SIFT, Polyphen-2, and CADD) and homozygous deleterious SNVs in the two data sets. (TIF)

S3 Fig Sharing of deleterious SNVs of 1000 Genomes PJL and ExAC SAS with other continental populations of their respective datasets. In both cases, sharing was observed in a descending order EUR>AMR>AFR>EAS>FIN. Notably, the proportion of sharing deleterious SNVs with European populations was greater for Mendelian and congenital CVDs than for common CVDs.
(TIF)

S4 Fig Violin plots to compare the density of predicted deleterious SNVs between Pakistani population versus all five groups of 1000 Genomes Project. The thickness of violins is proportional to the number of variants corresponding to that derived allele frequency (DAF). The box plots inside the violins showing the median values of DAF.
(TIF)

S5 Fig Based on Weir and Cockerham FST values, comparison of the proportions of moderately (FST 0.05–0.15), highly (FST 0.15–0.25), and severely (FST > 0.25) differentiated deleterious SNVs and all the SNVs in genes harboring these deleterious SNVs. The proportion of moderately differentiated SNVs is higher for deleterious SNVs when compared Pakistani population with all 25 populations of 1000 Genomes Project.
(TIF)

S6 Fig Comparison of the rare (AF < 0.5%), low (0.5% ≤ AF ≤ 5.0%), and common (AF > 5.0%)-allele frequency SNVs within the deleterious SNVs pool and total SNVs pool in genes of CVDs. The difference in the proportions of ‘rare variants’ within two categories i.e. total and deleterious, can be observed in each data set.
(TIF)

S1 Table The genes of common, Mendelian, and congenital CVDs analyzed in this study. (XLSX)

S2 Table ClinVar’s pathogenic, and likely pathogenic variants found in two datasets i.e., 1000 Genomes Project PJL and ExAC SAS. (XLSX)

S3 Table Predicted deleterious variants shared with other continental populations. (XLSX)

S4 Table Loss of Function variants found from ExAC SAS dataset. (XLSX)

S5 Table Deleterious SNVs of Pakistani population which are highly and severely differentiated from global populations of 1000 Genomes Project. It is note-worthy that two severely differentiated SNVs (rs560826688 and rs563254260) are both related to hypertension.
(DOCX)
