# Supplementary figures and images for: Estimating the mutational load for cardiovascular diseases in Pakistani population
Source: PLoS One. 2018 Feb 8;13(2):e0192446. doi: 10.1371/journal.pone.0192446 (PMC5805289; doi:10.1371/journal.pone.0192446)

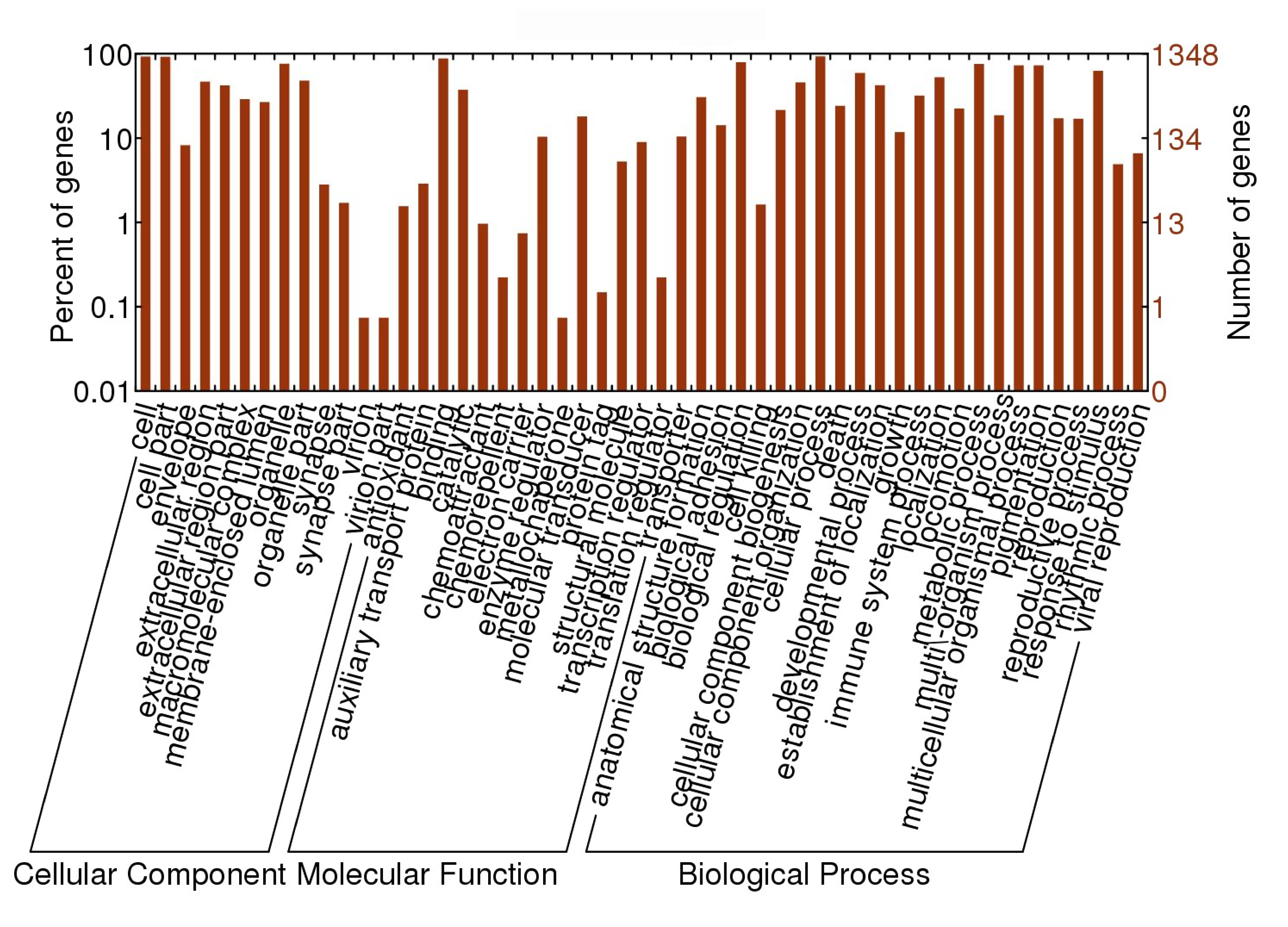

Supplement: S1 Fig — (TIF) [file pone.0192446.s002.tif]

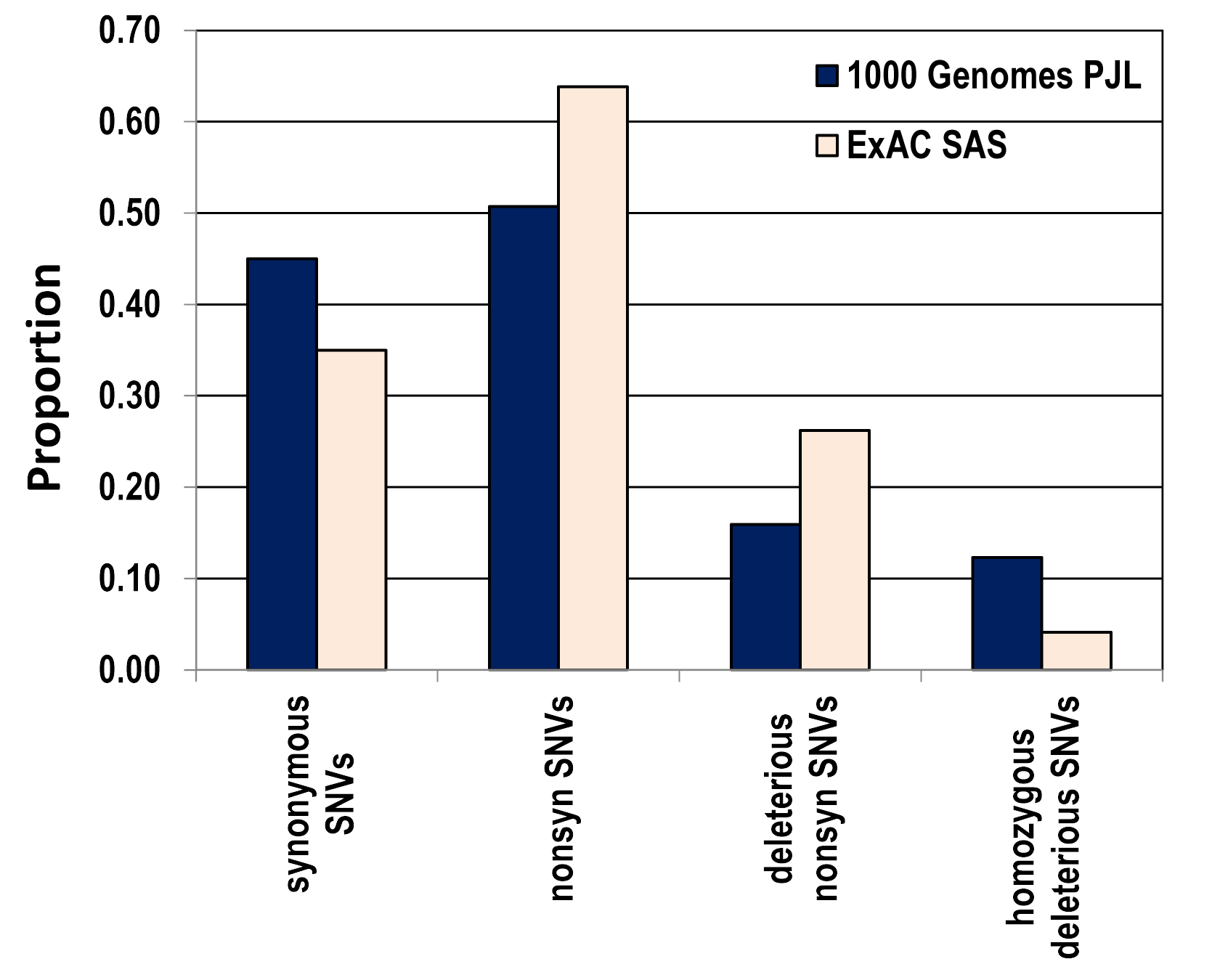

Supplement: S2 Fig — (TIF) [file pone.0192446.s003.tif]

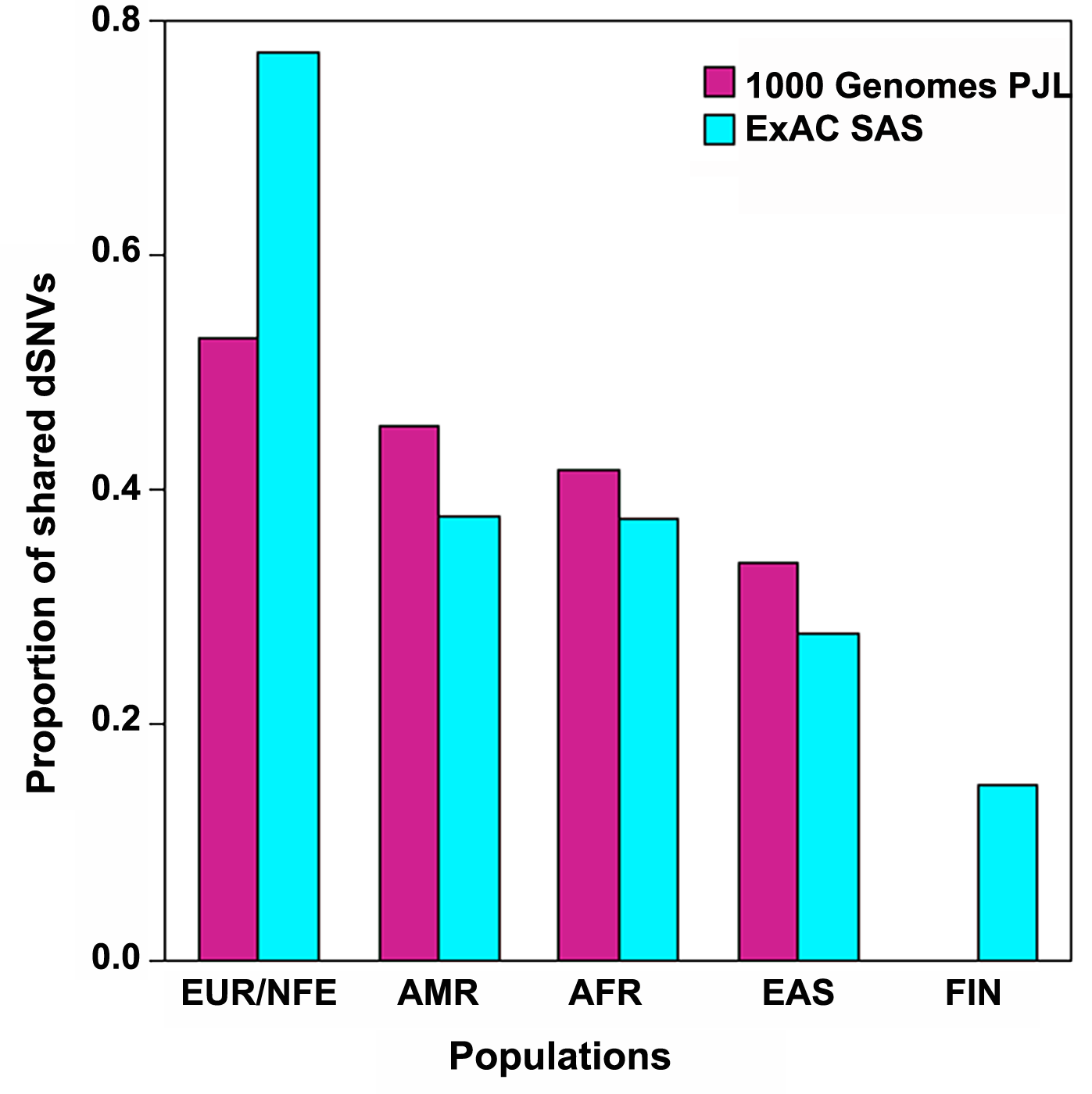

Supplement: S3 Fig — In both cases, sharing was observed in a descending order EUR>AMR>AFR>EAS>FIN. Notably, the proportion of sharing deleterious SNVs with European populations was greater for Mendelian and congenital CVDs than for common CVDs. (TIF) [file pone.0192446.s004.tif]

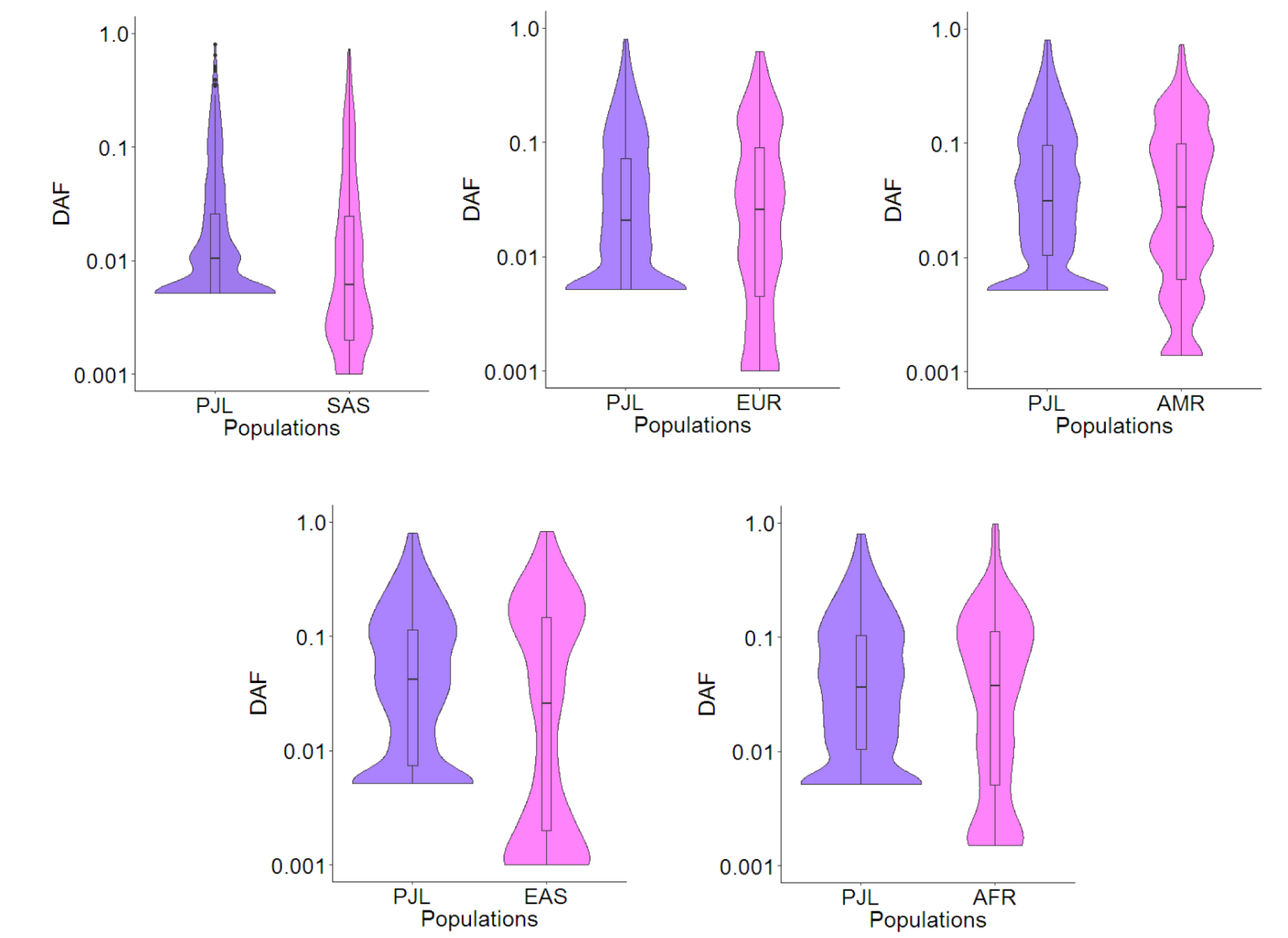

Supplement: S4 Fig — The thickness of violins is proportional to the number of variants corresponding to that derived allele frequency (DAF). The box plots inside the violins showing the median values of DAF. (TIF) [file pone.0192446.s005.tif]

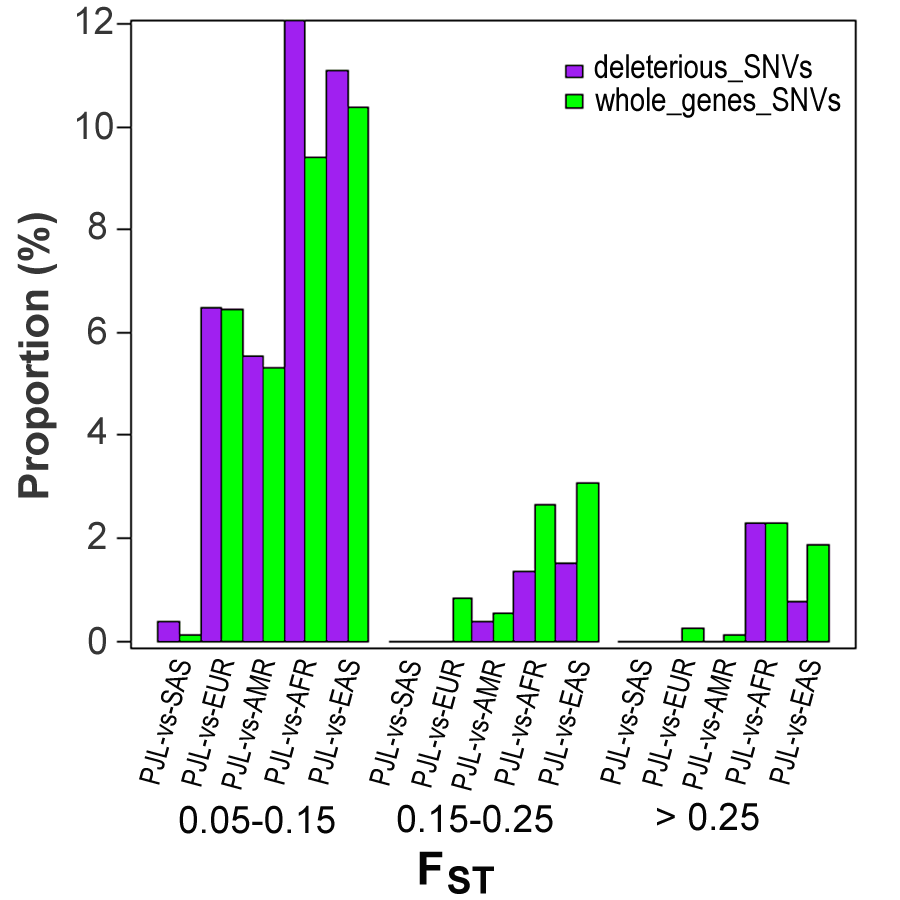

Supplement: S5 Fig — The proportion of moderately differentiated SNVs is higher for deleterious SNVs when compared Pakistani population with all 25 populations of 1000 Genomes Project. (TIF) [file pone.0192446.s006.tif]

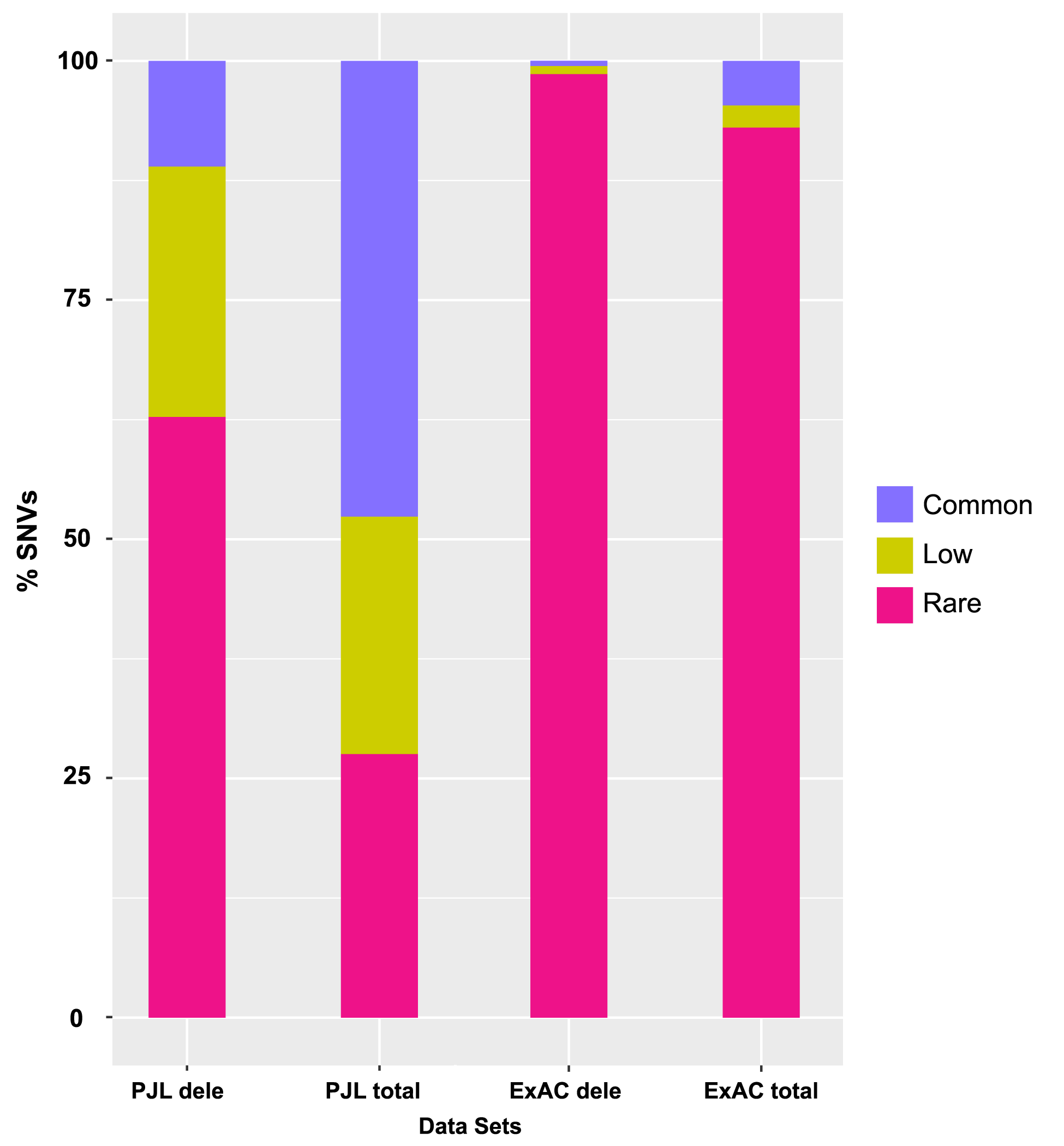

Supplement: S6 Fig — The difference in the proportions of ‘rare variants’ within two categories i.e. total and deleterious, can be observed in each data set. (TIF) [file pone.0192446.s007.tif]
