# Supplementary material for: Estimating the mutational load for cardiovascular diseases in Pakistani population
Source: PLoS One. 2018 Feb 8;13(2):e0192446. doi: 10.1371/journal.pone.0192446 (PMC5805289; doi:10.1371/journal.pone.0192446)
Supplement: S5 Table — It is note-worthy that two severely differentiated SNVs (rs560826688 and rs563254260) are both related to hypertension. (DOCX) [file pone.0192446.s012.docx]

| **CHR** | **POS** | **ID** | **REF** | **ALT** | **Gene** | **Global  Frequency** | **PJL Frequency** | **F_ST_** | **Disease** |
| --- | --- | --- | --- | --- | --- | --- | --- | --- | --- |
| 6 | 44274073 | rs151044424 | C | A | AARS2 | 0.000599042 | 0.015625 | 0.171381 | hypertrophic cardiomyopathy |
| 7 | 92731317 | rs577145375 | A | G | SAMD9 | 0.000599042 | 0.015625 | 0.171381 | atherosclerosis |
| 11 | 35240875 | rs376536014 | G | T | CD44 | 0.003793930 | 0.041667 | 0.172107 | aneurysm |
| *11* | *68192737* | *rs560826688* | *G* | *T* | *LRP5* | *0.001198080* | *0.03125* | *0.297321* | *hypertension* |
| 14 | 64678793 | rs532495528 | G | A | SYNE2 | 0.000599042 | 0.015625 | 0.170082 | dilated cardiomyopathy, Long QT syndrome |
| 14 | 74974786 | rs549001156 | C | A | LTBP2 | 0.000599042 | 0.015625 | 0.171381 | Ventricular septal defect |
| 15 | 67008780 | rs532621952 | C | G | SMAD6 | 0.000599042 | 0.015625 | 0.171381 | Aortic valve disease 2 |
| *17* | *1680660* | *rs563254260* | *C* | *G* | *SERPINF1* | *0.000998403* | *0.026042* | *0.259398* | *hypertension* |
| 19 | 39410407 | rs555119979 | G | A | SARS2 | 0.000798722 | 0.020833 | 0.217625 | hypertension |
| 21 | 38877614 | rs575017348 | T | C | DYRK1A | 0.000798722 | 0.020833 | 0.217625 | heart failure |

**Table S5:** Deleterious SNVs of Pakistani population which are highly and severely differentiated from global populations of 1000 Genomes Project. It is note-worthy that two severely differentiated SNVs (rs560826688 and rs563254260) are both related to hypertension.
